# Sperm IZUMO1-Dependent Gamete Fusion Influences Male Fertility in Mice

**DOI:** 10.3390/ijms20194809

**Published:** 2019-09-27

**Authors:** Takako Saito, Ikuo Wada, Naokazu Inoue

**Affiliations:** Department of Cell Science, Institute of Biomedical Sciences, School of Medicine, Fukushima Medical University, 1 Hikarigaoka, Fukushima City, Fukushima 960-1295, Japan; tsaito@fmu.ac.jp (T.S.); iwada@fmu.ac.jp (I.W.)

**Keywords:** spermatozoon, IZUMO1, biomarker, male fertility

## Abstract

Sperm–egg fusion is accomplished through the interaction of a specific set of membrane proteins in each gamete: sperm IZUMO1 and oocyte JUNO. Recently, we found that alternative splicing of the *Izumo1* gene generates a novel IZUMO1 isoform (IZUMO1_v2). Here, we obtained four mouse lines, having graded different levels of IZUMO1 protein by combining an original IZUMO1 (IZUMO1_v1) knockout with IZUMO1-null (both IZUMO1_v1 and _v2 disrupted) genetic background, in order to determine how the quantity of IZUMO1 influences male fertility. Subsequently, we clarified that the signal intensity from two quantitative assays, western blot and immunostaining analyses with a monoclonal antibody against mouse IZUMO1, were strongly correlated with average litter size. These results suggest that evaluating IZUMO1 protein levels is useful for predicting fecundity, and is a suitable test for male fertility.

## 1. Introduction

Fertilization is a complex phenomenon containing multiple essential events. Ejaculated spermatozoa undergo physiological changes, such as capacitation and hyperactivation, and morphological modification, acrosome reaction (AR), prior to reaching the oocyte. Finally, only acrosome-reacted spermatozoa are capable of proceeding to gamete fusion, the final step of fertilization [1,2]. Some cases of male sterility are caused by defects in male factors that affect sperm migration though the utero-tubal junction [3,4]. The sperm head is divided into an acrosomal cap, a post-acrosomal region and an equatorial segment, which is the site that contacts the oocyte and triggers gamete fusion [1]. During spermiogenesis, an acrosomal granule derived from multiple small proacrosomal vesicles partially covers the anterior sperm nucleus, eventually forming a cap-like acrosome. Simultaneously, many proteins, including those indispensable for accomplishing fertilization, are accumulated and distributed to their proper locations. Once these proteins are unable to function, males experience pathological conditions such as azoospermia, asthenospermia and teratozoospermia, along with sterility. These pathological states can be easily detected by semen analysis, such as sperm morphology and the motility test. Semen analysis is performed routinely, and has been commonly used for clinical examinations and animal breeding systems. However, in some cases of male infertility, conventional tests reveal no apparent abnormalities, indicating that the current methods of semen quality evaluation are limited. Therefore, the development of a novel methodology for the prognosis and diagnosis of male fertility is necessary.

Prior to the implementation of assisted reproductive technology (ART), including conventional artificial insemination (AI), in vitro fertilization (IVF) or intracytoplasmic sperm injection (ICSI), acrosomal proteins were considered to be useful biomarkers for evaluating sperm quality. Indeed, the human sperm acrosome associated (SPACA) 1 protein staining pattern with specific antibodies, which can be classified into three grades (strong, intermediate or faint, and no signal), was significantly correlated with developmental rates of embryos to blastocysts [5]. Interestingly, in bovine models, it has been suggested that a loss of tyrosine-phosphorylation of SPACA1-including proteins in spermatozoa is also strongly linked to AI subfertility [6]. 

Another acrosomal protein IZUMO1, an indispensable factor for gamete fusion, consists of an IZUMO domain, a *N*-glycosylated immunoglobulin-like domain, and a transmembrane region with a short cytoplasmic tail [7,8,9]. The IZUMO domain includes a binding platform to the IZUMO1 receptor JUNO, which is a glycosylphosphatidylinositol (GPI)-anchored oolemma protein [10,11,12]. Male mice lacking IZUMO1 are completely infertile due to sperm–egg fusion failure, even though their spermatozoa have a normal shape and motility, along with the ability to penetrate the zona pellucida, which is an extracellular matrix surrounding the oocyte [7]. Given the involvement of IZUMO1 in fertilization, its evaluation can be used for fecundity prediction.

We recently found a novel IZUMO1 isoform (IZUMO1_v2) that is encoded from a different exon (exon 1b) of the *Izumo1* gene through alternative splicing. IZUMO1_v2 possesses identical mature protein and functional properties as the original IZUMO1 (IZUMO1_v1); however, an IZUMO1_v1-specific knockout mouse carried only 19% of IZUMO1 protein compared to wild-type mice, and still retained male fertility. This also suggested that a minimal amount of IZUMO1 is sufficient for completing the sperm–egg fusion [13]. However, the minimum quantity of IZUMO1 molecules required for gamete fusion is still unknown.

In the current study, to elucidate how male fertility is affected by IZUMO1 quantity, we obtained four male mouse lines displaying different graded IZUMO1 expression levels, and investigated the correlation between the amount of IZUMO1 protein and the final outcome of fertilization; litter size. Our data suggest that IZUMO1 is a useful biomarker for male fertility. 

## 2. Results

### 2.1. Comparison of IZUMO1 Protein Quantity Among Each Mouse Line

**Figure 1:**
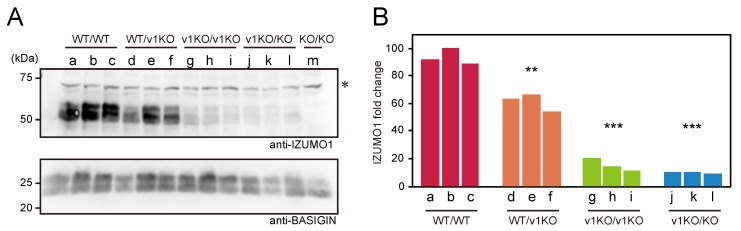
Evaluation of protein levels for IZUMO1 analyzed by western blotting. (**A**) Western blotting analysis with 2.2 μg/mL of monoclonal anti-IZUMO1 antibody (Mab18). Sperm lysates (30 μg) were applied onto each lane from an individual derived from five mouse lines (WT/WT; a–c, WT/v1KO; d–f, v1KO/v1KO; g–i, v1KO/KO; j–l and KO/KO; m). The alphabetical letters indicate individual specimens. BASIGIN is used as an internal control. The asterisk indicates non-specific bands. (**B**) Relative quantification for IZUMO1 protein normalized using BASIGIN. The color scheme is as follows: WT/WT; red, WT/v1KO; orange, v1KO/v1KO; light green, v1KO/KO; blue. Significant differences: ** *p* < 0.01 and *** *p* < 0.001 (Student’s *t*-test) compared to WT/WT.

Previously, we reported that IZUMO1 knockout (KO/KO; both IZUMO1_v1 and _v2 are disrupted) male mice are sterile, whereas IZUMO1_v1 specific knockout (v1KO/v1KO) male mice still carrying functional IZUMO1_v2 are fertile and have a smaller litter size compared to wild-type mice [7,13]. In order to elucidate how IZUMO1 protein quantity affects male fertility, we generated four types of mice carrying different protein levels of IZUMO1 (Table 1) by crossing IZUMO1 knockout, IZUMO1_v1 knockout, and wild-type (WT/WT) mice. Initially, we performed western blot analysis with an IZUMO1 monoclonal antibody (Mab18), using sperm BASIGIN as a normalizing protein to compare the amount of IZUMO1 protein in the spermatozoa of four mouse lines (Figure 1A). As a result, all IZUMO1 proteins were detectable, except for IZUMO1 knockout mice (KO/KO), although there was a significant difference in each protein level (Figure 1A,B). The average IZUMO1 protein levels of both of v1KO/v1KO and v1KO/KO spermatozoa were 16% and 11% of those of the wild-type spermatozoa, respectively (Figure 1B). 

### 2.2. Immunostaining of Spermatozoa from Each Mouse Line

**Figure 2:**
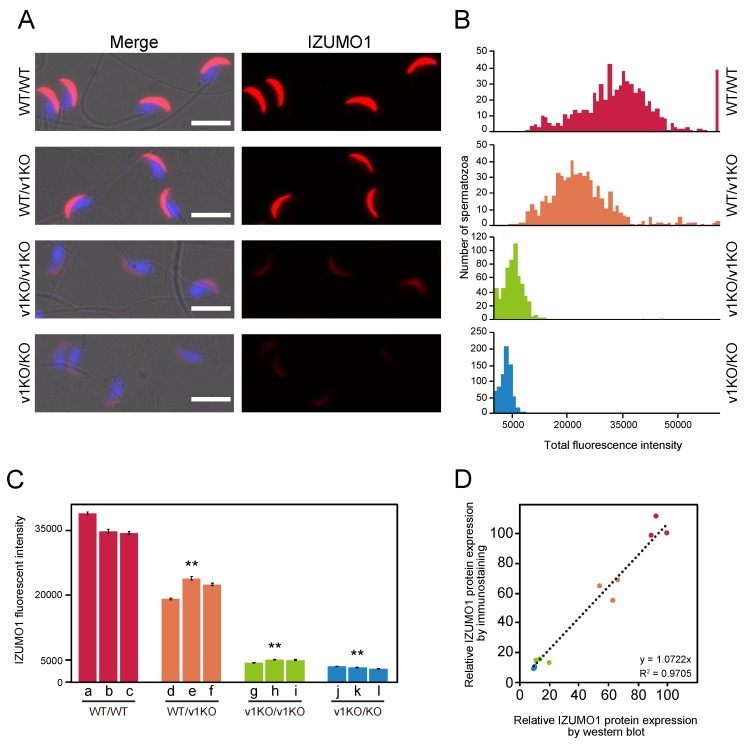
Evaluation of protein levels for IZUMO1 analyzed by immunostaining. (**A**) Immunostaining of spermatozoa with 0.5 μg/mL of monoclonal anti-IZUMO1 antibody (Mab34)-conjugated to Alexa Fluor 546 (red). Sperm nuclei are stained with Hoechst 33342 (blue). Scale bar is 10 μm. (**B**) Histograms of normalized fluorescence intensity obtained from spermatozoa shown by immunostaining. (**C**) Average of fluorescence intensity from individual spermatozoa. Each bar represents an individual spermatozoa (each alphabetical letter corresponds to Figure 1A.) from four mouse lines. Significant differences: ** *p* < 0.01 (Student’s *t*-test) compared to WT/WT. (**D**) Comparison of relative IZUMO1 protein values obtained via two methods. Each specimen derivation between the two methods is identical. The color scheme is the same as that in Figure 1B.

To assess the amount of IZUMO1 protein *in situ*, spermatozoa from each mouse line were stained with an anti-IZUMO1 monoclonal antibody (Mab34), and the population of each spermatozoon’s fluorescence intensity was then measured. IZUMO1 relocates from the acrosomal cap region to the equatorial segment during AR [7,14,15]. Therefore, we employed fresh sperm samples before AR for better fluorescence signal quantification because acrosome intact spermatozoa are uniform fractions of staining patterns. Essentially, identical IZUMO1 localization in the acrosomal cap region was observed in all sets of spermatozoa; however, the IZUMO1 fluorescence of the v1KO/v1KO and v1KO/KO spermatozoa were dimmer than that in the WT/WT spermatozoa (Figure 2A).

### 2.3. Measurement of IZUMO1 Protein by Immunostaining 

To perform the statistical analysis of the amount of IZUMO1 protein expressed in a spermatozoon, we prepared Z-stacks of images captured using a 20× objective lens (NA0.75). The immuno-fluorescence signals of IZUMO1 for all spermatozoa were collected from an optical space of ~10 μm thickness, and the intensity of each cell at the maximum intensity projection was determined. The intensity histogram of each mouse line represented the distribution of total IZUMO1 expression level (Figure 2B). The histograms of IZUMO1 in the WT/v1KO, v1KO/v1KO and v1KO/KO mice were shifted to the left compared to the WT/WT mice. The WT/WT mice had a slightly broader distribution with the left shoulder, which may suggest failure of translation from the authentic initiation codon in some cells. When the average IZUMO1 signal in each spermatozoa from three mice was also compared, the expression levels of IZUMO1 were significantly different among the mouse lines (Figure 2C), suggesting that the IZUMO1 quantity very much depends on the genetic background. This was also consistent with our western blot data (Figure 1).

### 2.4. Comparison of IZUMO1 Expression Levels Using Different Two Methods

To corroborate the output from both methodologies (western blot and immunostaining analyses), we performed a direct comparison between the two. The integrated intensity of the bands in the western blot analysis (X-axis) were plotted against the average intensities of immunostaining for each individual (Y-axis), implying that IZUMO1 quantities determined by western blotting and immunostaining were well-correlated (*R^2^* = 0.9705, Figure 2D).

### 2.5. Average Litter Size from Each Mouse Line

**Figure 3:**
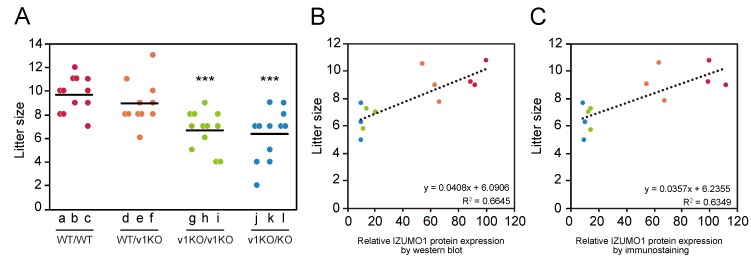
Comparison between litter size and IZUMO1 protein levels. (**A**) Litter size of mice mated with B6D2F1 female mice. Each lane represents an individual score from the four mouse lines. Each alphabetical letter corresponds to the same male individuals as in Figure 1A. The average litter size of each mouse line (total number of pups/total number of births) is indicated by the black bar. Significant differences: *** *p* < 0.001 (Student’s *t*-test) compared to WT/WT. Correlation of litter size and IZUMO1 expression levels determined with western blot (**B**) and immunostaining (**C**). The color scheme is the same as that in Figure 1B.

Male mice of different IZUMO1 genetic backgrounds were caged with wild-type females to elucidate how IZUMO1 protein levels affect male fertility. In order to exclude the instability of female factors, such as uterine capacity, ovulation rate and embryonic development, we used wild-type B6D2F1 female mice because of their genetic uniformity, stable ovulation and high fertilization rate [16]. The average litter sizes (total number of pups/total number of births) of the mouse lines were WT/WT: 9.67 ± 0.41, WT/v1KO: 8.91 ± 0.54, v1KO/v1KO: 6.67 ± 0.45, and v1KO/KO: 6.33 ± 0.59 (Figure 3A). Significant differences were observed between WT/WT and v1KO/v1KO (*p* = 0.0001), which was consistent the results of our previous report [13], and also WT/WT and v1KO/KO (*p* = 0.0003). However, there was no significant difference in litter size between v1KO/v1KO and v1KO/KO (*p* = 0.67). Together with the quantification data of the IZUMO1 protein (Figure 1 and Figure 2), these results showed that IZUMO1 protein levels apparently influenced the litter size.

### 2.6. Correlation of IZUMO1 Protein Amount with Litter Size

When compared to those of wild-type mice, the average litter sizes of the v1KO/v1KO and v1KO/KO mice were significantly decreased, although the litter sizes of the WT/v1KO mice were not (Figure 3A). Taking advantage of the observation of IZUMO1 signal intensities obtained via western blot or immunostaining analysis with this data, male fertility could be evaluated. In Figure 3B,C, the X- and Y-axes represent relative IZUMO1 protein levels and average litter size, respectively. The two methodologies revealed that fecundity was positively correlated with IZUMO1 protein levels. These data clearly demonstrated that male fertility is affected by the amount of IZUMO1 protein.

## 3. Discussion

Although a semen test is important for determining the diagnosis and sperm quality, information from conventional sperm analysis, such as sperm morphology and the motility test, is limited. For example, it is well-known that absence or abnormal expression of acrosomal proteins, which are involved in fertilization, cause infertility [3,4]. However, some defects are not detectable through conventional tests because deficiencies of the essential acrosomal proteins such as IZUMO1 or SPACA6 cause serious male infertility in mice without causing morphological or motility change in spermatozoa [7,17]. Thus, in such cases, it is more practical to perform AI or IVF after the evaluation of the acrosomal proteins. Although ICSI bypasses the gamete fusion process by direct injection of single spermatozoon into the oocyte, the assessment of acrosomal proteins can help to select the very best spermatozoa.

In the present study, we demonstrated that the signal intensity of IZUMO1 from western blot and immunostaining analyses were strongly correlated with male fertility. As the immunostaining method requires only a minimal amount of semen, this could be widely applicable. It should be noted that the IZUMO1 localization, as evaluated from the procedure, is valuable for identifying superior spermatozoa. Currently, AI has been generally performed in animal industries to produce a large number of offspring with excellent genetic traits [6,18]. Higher AI efficiency is economically important, and larger litter size breeds are preferred. However, since cryopreserved bull spermatozoa, which generally have a low fertilization rate compared to freshly ejaculated spermatozoa, often lose acrosomes and the IZUMO1 protein [19], direct examination of the IZUMO1 protein should be useful to test semen quality. In addition, considering that IZUMO1 is conserved among broad species in vertebrates [20], it may be possible to apply this method to other animals.

Currently, it remains unknown how exactly IZUMO1 triggers sperm–egg fusion. The IZUMO1-JUNO recognition system is insufficient for the completion of cell–cell fusion as IZUMO1-overexpressing cultured cells are incapable of fusion with the oocyte, even with occurrence of a cell–oocyte interaction [21,22,23]. Moreover, cluster of differentiation 9 (CD9) knockout females have been reported to exhibit a severely reduced litter size and fusion ability [24,25,26], and it was also reported that CD9 deficient oocytes still retain recombinant IZUMO1 binding ability [21]. Thus, there must be multiple steps associated with the interaction of IZUMO1-JUNO, and uncharacterized molecules are likely to be involved during sperm–egg fusion [23]. Therefore, such novel proteins involved in the IZUMO1-JUNO recognition system will also become candidates for predicting fecundity. In addition, in order to understand the detailed function of IZUMO1_v2, the influence on fertility of male mice solely lacking IZUMO1_v2 needs to be elucidated in the near future.

Because the amount of IZUMO1 is critical for male fertility, as described in the current study, it should be noted that complementation of IZUMO1 may be suitable for infertility treatments and, conversely, inhibition of IZUMO1 is likely to be useful as a contraceptive strategy. Indeed, IZUMO1 has been used as a target for novel non-hormonal contraception [27]. Female mice displayed a significant reduction of fertility after vaccination of IZUMO1 protein or cDNA [28,29,30,31,32].

## 4. Materials and Methods 

### 4.1. Mice

The B6D2F1 mice were purchased from Japan SLC Inc. (Hamamatsu, Japan), and the IZUMO1 KO and IZUMO1_v1 KO mice were produced as previously described [7,13]. All animal studies were approved by the Animal Experiments Committee of Fukushima Medical University, Japan (approval number: 30046, 1 April 2018), and performed under the guidelines and regulations of Fukushima Medical University.

### 4.2. Genotyping 

Mouse tail-tip DNA and polymerase chain reaction (PCR) were used for genotyping. PCR was carried out using Ex Taq (Takara bio, Kusatsu, Japan). IZUMO1 KO and IZUMO1_v1 KO alleles were detected by previously-described specific primer sets [7,13].

### 4.3. Measurement of Litter Size

The litter size experiment was conducted on three males (>10 weeks old) from each mouse line, which were mated with B6D2F1 females (>8 weeks old) four times. The litter size per female was counted on the first day of birth. 

### 4.4. Western Blot Analysis of Spermatozoa

After measuring litter size, the spermatozoa were individually collected from the cauda epididymis and vas deferens of the male mice. The sperm proteins were solubilized in PBS with 1% Triton-X100 and a protease inhibitor cocktail (FUJIFIM Wako Pure Chemical Corporation, Osaka, Japan) for 1 h on ice. After centrifuge at 17,000× *g* for 30 min, 30 μg of sperm proteins were separated using SDS-PAGE and transferred to a PVDF membrane (Merck Millipore, Darmstadt, Germany). The membranes were probed using primary antibodies (anti-IZUMO1 antibody [Mab18] [23] and anti-BASIGIN antibody [sc-9757: Santa Cruz Biotechnology, Santa Cruz, CA, USA]) followed by secondary antibodies conjugated to HRP (Jackson ImmunoReserch Laboratories, West Grove, PA, USA). Chemiluminescence reactions were performed with ECL Prime (GE Healthcare Life Sciences, Chicago, IL, USA).

### 4.5. Fluorescence Imaging for Spermatozoa

The spermatozoa were washed with PBS and centrifuged at 900× *g* for 5 min twice, air dried on slide glasses at 42 °C, fixed with 4% paraformaldehyde in PBS for 5 min at room temperature, and washed three times with PBS. The slide glasses were blocked for 1 h with 0.1% Tween / 5% BSA in PBS, and incubated in the dark with 0.5 μg/mL anti-IZUMO1 antibody (Mab34-Alexa Fluor 546) [21] and 1.0 μg/mL Hoechst 33342 (Sigma-Aldrich, St. Louis, MO, USA) for 2 h at room temperature. After washing with PBS, the spermatozoa were observed under an A1 confocal microscope (Nikon, Tokyo, Japan) with a 60× objective lens (NA 1.27) and Z-stack images were acquired with a 20× objective lens (NA 0.75).

### 4.6. Image and Statistical Analyses

The maximum intensity Z-projection of all Z-stack images was performed with the open source software Fiji [33]. The correlation between the immunostaining results and the western blotting results, as well as that between litter size and IZUMO1 protein levels, were tested using Pearson’s correlation coefficient.

## Figures and Tables

**Table 1:** Four different mouse lines of *Izumo1* gene disruption. Note that the WT, v1KO and KO mouse lines have wild-type, IZUMO1_v1 knockout, and both IZUMO1_v1 and v2 knockout alleles, respectively. Corresponding to their genetic background, each allele is expressed as a plus or minus.

	WT/WT	WT/v1KO	v1KO/v1KO	v1KO/KO
IZUMO1_v1	+/+	+/-	-/-	-/-
IZUMO1_v2	+/+	+/+	+/+	+/-
